# Recent Advances in Pancreato-Biliary Endoscopic Intervention: How to Resolve Unmet Needs in Pancreato-Biliary Diseases Endoscopically

**DOI:** 10.3390/jcm11133637

**Published:** 2022-06-23

**Authors:** Hiroyuki Isayama, Shigeto Ishii, Ko Tomishima, Toshio Fujisawa

**Affiliations:** Department of Gastroenterology, Graduate School of Medicine, Juntendo University, Tokyo 113-8421, Japan; sishii@juntendo.ac.jp (S.I.); tomishim@juntendo.ac.jp (K.T.); t-fujisawa@juntendo.ac.jp (T.F.)

Various procedures are available for pancreato-biliary (PB) endoscopic interventions. Furthermore, multiple guidelines have been published, and various accessories are commercially available; however, there are many unmet needs in the endoscopic management of PB diseases [1,2]. Guidewires are indispensable for endoscopic procedures; however, there remains no ideal guidewire. Alternative efforts toward developing and evaluating devices for endoscopic procedures have been critical in increasing the efficiency of our procedure [3]. Further development of strategies and devices are needed to resolve the unmet needs in this field.

Common bile duct stones can be managed endoscopically, which is considered a standard procedure. However, it is unclear what the remaining unmet needs are for the endoscopic management of common bile duct stones. Endoscopic sphincterotomy (EST) was the standard procedure for PB endoscopic treatment, but it is difficult for beginners, and pancreatitis and bleeding after EST are difficult to manage and can be fatal [4]. Performing EST to open the papilla is complicated, and retraction of common bile duct stones can be problematic even with full-length EST. EST followed by balloon dilation (ESBD) to open the papilla to retract common bile duct stones can be performed by beginner endoscopists because of the small incision with balloon dilation [3], facilitating subsequent stone retraction. Bleeding is less common with ESBD than with EST because of the small incision, and pancreatitis is less common than endoscopic papillary balloon dilation (EPBD) because of the division of the pancreas and bile duct orifice [5]. Management of patients with common bile duct stones who cannot tolerate prolonged endoscopic procedures is difficult. Long-term placement of plastic stents without stone removal is an alternative treatment option [6]. However, multiple large stones can be retrieved efficiently by large-balloon papillary dilation, endoscopic sphincterotomy followed by large-balloon dilation (ESLBD), and endoscopic papillary large-balloon dilation (EPLBD) [5,7]. Removal of common bile duct stones by new modalities is a safer means of patient management. However, no evidence is available, and thus a prospective comparative study is needed.

Pancreatoduodenectomy, surgical papillectomy, and endoscopic papillectomy can be used to resect papillary tumors. Invasive modalities result in more complete resection but have higher complication rates [8]. However, for benign lesions and carcinoma in situ, a less invasive modality is needed. Endoscopic papillectomy does not always achieve complete resection and is risky in terms of bleeding and perforation [9]. Additionally, cannulation from resected lesions is problematic. Following prevention of bleeding, the resected area is closed with clips. Hemostatic procedures are stressful for endoscopists. Endoscopic submucosal dissection maintains the safety margin for complete resection [10] but is time consuming. More efficient and safer endoscopic papillectomy procedures, as well as prospective studies, are needed.

Endoscopic stenting is a standard procedure, but the ideal strategy and stent, especially in the management of hilar malignant biliary obstruction, are unclear [11]. A biliary plastic stent is placed above the papilla to prevent reflux of duodenal juice and contents, with the aim of reducing the incidence of recurrent biliary obstruction (RBO) and subsequent long time to RBO. This stent was developed by a Japanese endoscopist [12,13,14] but has not been evaluated prospectively or in a comparative study. A prospective single-arm multicenter study evaluated the feasibility of an inside stent [15]. The inside stent showed a long time to RBO in cases of benign biliary stricture (BBS) and superiority to conventional plastic stents in malignant cases. In the Japanese clinical practice guidelines for biliary cancer, plastic stents and self-expandable metallic stents are recommended equally for the management of unresectable hilar cholangiocarcinoma [16]. The difficulty of re-intervention because of an occluded stent is a problem with uncovered self-expandable metal stents (SEMSs), and exchangeability is important for hilar cholangiocarcinoma because of prolonged survival due to effective chemotherapy [17].

Endoscopic management of BBS has changed markedly in the last decade. Endoscopic procedures aim to both relieve symptoms and resolve the stricture [18]. To dilate the BBS, placement of multiple plastic stents and fully covered SEMSs is effective [19,20]. Fully covered SEMSs are effective for BBS via the percutaneous and anastomosis routes, followed by endoscopic ultrasonography (EUS)-guided biliary drainage [21]. Bilioenteric anastomosis stricture hampers approaching the anastomotic site via an endoscope. The percutaneous and transluminal routes are promising but are not ideal for inserting multiple catheters. Fully covered SEMSs are recommended for such cases, despite the lack of an established removal method. Free insertion of stents by various routes and to a variety of locations may be beneficial for both patients and endoscopists [22].

The initial indication for interventional EUS (IV-EUS) was management of peri-pancreatic fluid collection including pseudocysts and walled-off necrosis [23]. EUS-guided drainage/anastomosis (EUS-D/A) is effective, but infected necrotic tissues and dead space including infected liquid confined in necrotic tissues cannot be drained by simple drainage. Necrosectomy enables retrieval of necrotic tissues and opening of the dead space [24]. Currently, there are many guidelines for the management of walled-off necrosis after severe pancreatitis [25,26]; however, the procedure is inefficient and risky and may be fatal.

EUS-guided rendezvous enables biliary access when conventional cannulation fails [27]. According to the scope position, there are three routes: transgastric, transduodenal short scope position, and transduodenal long scope position [28,29]. Each has its advantages and disadvantages, and no standard strategy has been established. Matsubara et al. proposed a new algorithm for selecting the approach route [30]; the hitch-and-ride technique they developed reduces the difficulty of EUS-guided rendezvous [31].

A stent is key for treatment success and a good prognosis. Transluminal drainage/anastomosis stents (T-DAS) for IV-EUS required various abilities, insertion, drainage, prevention of leakage and migration. Lee et al. reviewed conventional stents and dedicated T-DASs and described their functions in interventional EUS procedures [32]. Prevention of migration is important for IV-EUS drainage [27]. This fatal complication could be prevented by developing effective T-DASs but also by considering the patient’s status. Ochial et al. analyzed risk factors for stent migration in EUS-guided hepaticogastrostomy [33]. From their experience, there is both real migration and imminent migration, and the distance between the liver and gastric wall on computed tomography before the procedure was a risk factor. During the procedure, we aim to shorten this distance, but the gastric wall may return to the original position after treatment. In such cases, we select a >10 cm stent with >5 cm in the stomach [34]. Development of a covered SEMS with effective anchorage is needed for widespread use of EUS-guided hepaticogastrostomy.

After successful EUS-guided drainage/anastomosis, several procedures involving anastomosis may be performed. Endoscopic necrosectomy was reported by Seewald et al. [35]. EUS-D/A can create a route between non-adherent organs, facilitating endoscopic procedures. After EUS-D/A, stone and stricture management by cholangioscopy/pancreatotomy is possible in cases of biliary, gallbladder, and pancreatic drainage [36,37,38,39]. These are transluminal endoscopic procedures or endoscopic procedures through the anastomosis following EUS-D/A. After EUS-guided gallbladder drainage, stones are removed through the matured anastomosis, and cholecystectomy is not necessary.

In conclusion, various endoscopic procedures can be used to ameliorate symptoms. However, there are many unmet needs. Efforts to establish a strategy and develop new methods/devices will enhance the safety and efficacy of the procedure.

## References

[1] Adler D.G., Baron T.H., Davila R.E., Egan J., Hirota W.K., Leighton J.A., Qureshi W., Rajan E., Zuckerman M.J., Fanelli R. (2005). ASGE guideline: The role of ERCP in diseases of the biliary tract and the pancreas. Gastrointest. Endosc..

[2] Dumonceau J.-M., Andriulli A., Deviere J., Mariani A., Rigaux J., Baron T., Testoni P. (2010). European Society of Gastrointestinal Endoscopy (ESGE) Guideline: Prophylaxis of Post-ERCP Pancreatitis. Endoscopy.

[3] Ishii S., Fujisawa T., Isayama H., Asahara S., Ogiwara S., Okubo H., Yamagata H., Ushio M., Takahashi S., Okawa H. (2020). Clinical evaluation of a newly developed guidewire for pancreatobiliary endoscopy. J. Clin. Med..

[4] Buxbaum J.L., Fehmi S.M.A., Sultan S., Fishman D.S., Qumseya B.J., Cortessis V.K., Schilperoort H., Kysh L., Matsuoka L., Yachimski P. (2019). ASGE guideline on the role of endoscopy in the evaluation and management of choledocholithiasis. Gastrointest. Endosc..

[5] Ishii S., Isayama H., Ushio M., Takahashi S., Yamagata W., Takasaki Y., Suzuki A., Ochiai K., Tomishima K., Kanazawa R. (2020). Best procedure for the management of common bile duct stones via the papilla: Literature review and analysis of procedural efficacy and safety. J. Clin. Med..

[6] Sbeit W., Khoury T., Kadah A., Livovsky D.M., Nubani A., Mari A., Goldin E., Mahamid M. (2020). Long-term safety of endoscopic biliary stents for cholangitis complicating choledocholithiasis: A multi-center study. J. Clin. Med..

[7] Kogure H., Kawahata S., Mukai T., Doi S., Iwashita T., Ban T., Ito Y., Kawakami H., Hayashi T., Sasahira N. (2020). Multicenter randomized trial of endoscopic papillary large balloon dilation without sphincterotomy versus endoscopic sphincterotomy for removal of bile duct stones: MARVELOUS trial. Endoscopy.

[8] Heise C., Abou Ali E., Hasenclever D., Auriemma F., Gulla A., Regner S., Gaujoux S., Hollenbach M. (2020). Systematic review with meta-analysis: Endoscopic and surgical resection for ampullary lesions. J. Clin. Med..

[9] Vanbiervliet G., Strijker M., Arvanitakis M., Aelvoet A., Arnelo U., Beyna T., Busch O., Deprez P.H., Kunovsky L., Larghi A. (2021). Endoscopic management of ampullary tumors: European Society of Gastro-intestinal Endoscopy (ESGE) Guideline. Endoscopy.

[10] Takahara N., Tsuji Y., Nakai Y., Suzuki Y., Inokuma A., Kanai S., Noguchi K., Sato T., Hakuta R., Ishigaki K. (2020). A novel technique of endoscopic papillectomy with hybrid endoscopic submucosal dissection for ampullary tumors: A proof-of-concept study (with Video). J. Clin. Med..

[11] Lee T.H., Moon J.H., Park S.H. (2020). Biliary stenting for hilar malignant biliary obstruction. Dig. Endosc..

[12] Yazumi S., Yoshimoto T., Hisatsune H., Hasegawa K., Kida M., Tada S., Uenoyama Y., Yamauchi J., Shio S., Kasahara M. (2006). Endoscopic treatment of biliary complications after right-lobe living-donor liver transplantation with duct-to-duct biliary anastomosis. J. Hepato-Biliary-Pancreat. Surg..

[13] Uchida N., Tsutsui K., Ezaki T., Fukuma H., Kamata H., Kobara H., Matsuoka H., Kinekawa F., Aritomo Y., Yokoyama F. (2005). Estimation of the stent placement above the intact sphincter of Oddi against malignant bile duct obstruction. J. Gastroenterol..

[14] Tsujino T., Isayama H., Sugawara Y., Sasaki T., Kogure H., Nakai Y., Yamamoto N., Sasahira N., Yamashiki N., Tada M. (2006). Endoscopic management of biliary complications after adult living donor liver transplantation. Am. J. Gastroenterol..

[15] Kogure H., Kato H., Kawakubo K., Ishiwatari H., Katanuma A., Okabe Y., Ueki T., Ban T., Hanada K., Sugimori K. (2021). A prospective multicenter study of “inside stents” for biliary stricture: Multicenter evolving inside stent registry (MEISteR). J. Clin. Med..

[16] Nagino M., Hirano S., Yoshitomi H., Aoki T., Uesaka K., Unno M., Ebata T., Konishi M., Sano K., Shimada K. (2020). Clinical practice guidelines for the management of biliary tract cancers 2019: The 3rd English edition. J. Hepato-Biliary-Pancreat. Sci..

[17] Valle J., Wasan H., Palmer D.H., Cunningham D., Anthoney A., Maraveyas A., Madhusudan S., Iveson T., Hughes S., Pereira S.P. (2010). Cisplatin plus gemcitabine versus gemcitabine for biliary tract cancer. N. Engl. J. Med..

[18] Chathadi K.V., Chandrasekhara V., Acosta R.D., Decker G.A., Early D.S., Eloubeidi M.A., Evans J.A., Faulx A.L., Fanelli R.D., ASGE Standards of Practice Committee (2015). The role of ERCP in benign diseases of the biliary tract. Gastrointest. Endosc..

[19] Cote G.A., Slivka A., Tarnasky P., Mullady D.K., Elmunzer B.J., Elta G., Fogel E., Lehman G., McHenry L., Romagnuolo J. (2016). Effect of covered metallic stents compared with plastic stents on benign biliary stricture resolution: A randomized clinical trial. JAMA.

[20] Akbar A., Irani S., Baron T.H., Topazian M., Petersen B.T., Gostout C.J., Levy M.J., Gan I., Gluck M., Ross A. (2012). Use of covered self-expandable metal stents for endoscopic management of benign biliary disease not related to stricture (with video). Gastrointest. Endosc..

[21] Tomishima K., Ishii S., Fujisawa T., Ikemura M., Ushio M., Takahashi S., Yamagata W., Takasaki Y., Suzuki A., Ito K. (2021). Evaluation of the feasibility and effectiveness of placement of fully covered self-expandable metallic stents via various insertion routes for benign biliary strictures. J. Clin. Med..

[22] Dumonceau J.-M., Devière J., Delhaye M., Baize M., Cremer M. (1998). Plastic and metal stents for postoperative benign bile duct strictures: The best and the worst. Gastrointest. Endosc..

[23] Banks P.A., Bollen T.L., Dervenis C., Gooszen H.G., Johnson C.D., Sarr M.G., Tsiotos G.G., Vege S.S., Acute Pancreatitis Classification Working Group (2013). Classification of acute pancreatitis—2012: Revision of the Atlanta classification and definitions by international consensus. Gut.

[24] Baron T.H., DiMaio C.J., Wang A.Y., Morgan K.A. (2020). American Gastroenterological Association Clinical Practice Update: Management of Pancreatic Necrosis. Gastroenterology.

[25] Isayama H., Nakai Y., Rerknimitr R., Khor C., Lau J., Wang H.-P., Seo D.W., Ratanachu-Ek T., Lakhtakia S., Ang T.L. (2016). Asian consensus statements on endoscopic management of walled-off necrosis Part 1: Epidemiology, diagnosis, and treatment. J. Gastroenterol. Hepatol..

[26] Isayama H., Nakai Y., Rerknimitr R., Khor C., Lau J., Wang H., Seo D.W., Ratanachu-ek T., Lakhtakia S., Ang T.L. (2016). Asian consensus statements on endoscopic management of walled-off necrosis. Part 2: Endoscopic management. J. Gastroenterol. Hepatol..

[27] Dhir V., Bhandari S., Bapat M., Maydeo A. (2012). Comparison of EUS-guided rendezvous and precut papillotomy techniques for biliary access (with videos). Gastrointest. Endosc..

[28] Isayama H., Nakai Y., Kawakubo K., Kawakami H., Itoi T., Yamamoto N., Kogure H., Koike K. (2012). The endoscopic ultrasonography-guided rendezvous technique for biliary cannulation: A technical review. J. Hepato-Biliary-Pancreat. Sci..

[29] Kawakubo K., Isayama H., Sasahira N., Nakai Y., Kogure H., Hamada T., Miyabayashi K., Mizuno S., Sasaki T., Ito Y. (2013). Clinical utility of an endoscopic ultrasound-guided rendezvous technique via various approach routes. Surg. Endosc..

[30] Matsubara S., Nakagawa K., Suda K., Otsuka T., Isayama H., Nakai Y., Oka M., Nagoshi S. (2020). A proposed algorithm for endoscopic ultrasound-guided rendezvous technique in failed biliary cannulation. J. Clin. Med..

[31] Nakai Y., Isayama H., Matsubara S., Kogure H., Mizuno S., Hamada T., Takahara N., Nakamura T., Sato T., Takeda T. (2017). A novel “hitch-and-ride” deep biliary cannulation method during rendezvous endo-scopic ultrasound-guided ERCP technique. Endoscopy.

[32] Park S.W., Lee S.S. (2020). Which are the most suitable stents for interventional endoscopic ultrasound?. J. Clin. Med..

[33] Ochiai K., Fujisawa T., Ishii S., Suzuki A., Saito H., Takasaki Y., Ushio M., Takahashi S., Yamagata W., Tomishima K. (2021). Risk factors for stent migration into the abdominal cavity after endoscopic ultrasound-guided hepaticogastrostomy. J. Clin. Med..

[34] Nakai Y., Sato T., Hakuta R., Ishigaki K., Saito K., Saito T., Takahara N., Hamada T., Mizuno S., Kogure H. (2020). Long-term outcomes of a long, partially covered metal stent for EUS-guided hepaticogas-trostomy in patients with malignant biliary obstruction (with video). Gastrointest. Endosc..

[35] Seewald S., Groth S., Omar S., Imazu H., Seitz U., de Weerth A., Soetikno R., Zhong Y., Sriram P.V., Ponnudurai R. (2005). Aggressive endoscopic therapy for pancreatic necrosis and pancreatic abscess: A new safe and effective treatment algorithm (videos). Gastrointest. Endosc..

[36] James T.W., Baron T.H. (2018). Antegrade pancreatoscopy via EUS-guided pancreaticogastrostomy allows removal of obstructive pancreatic duct stones. Endosc. Int. Open.

[37] Suzuki A., Ishii S., Fujisawa T., Saito H., Takasaki Y., Takahashi S., Yamagata W., Ochiai K., Tomishima K., Isayama H. (2022). Efficacy and safety of peroral pancreatoscopy through the fistula created by endoscopic ultrasound–guided pancreaticogastrostomy. Pancreas.

[38] Dhir V., Isayama H., Itoi T., Almadi M., Siripun A., Teoh A.Y.B., Ho K.Y. (2017). Endoscopic ultrasonography-guided biliary and pancreatic duct interventions. Dig. Endosc..

[39] Takasaki Y., Ishii S., Shibuya T., Fujisawa T., Ushio M., Takahashi S., Ito K., Yamagata W., Suzuki A., Okahara K. (2021). Endoscopic ultrasound-guided antegrade procedures for managing bile duct stones in patients with surgically altered anatomy: Comparison with double-balloon enteroscopy-assisted endoscopic retrograde cholangiography (with video). Dig. Endosc..

